# The prognostic role of immune checkpoint markers programmed cell death protein 1 (PD-1) and programmed death ligand 1 (PD-L1) in a large, multicenter prostate cancer cohort

**DOI:** 10.18632/oncotarget.15817

**Published:** 2017-03-01

**Authors:** Nora Ness, Sigve Andersen, Mehrdad Rakaee Khanehkenari, Cecilie V. Nordbakken, Andrej Valkov, Erna-Elise Paulsen, Yngve Nordby, Roy M. Bremnes, Tom Donnem, Lill-Tove Busund, Elin Richardsen

**Affiliations:** ^1^ Department of Medical Biology, UiT The Arctic University of Norway, N-9037 Tromso, Norway; ^2^ Department of Clinical Medicine, UiT The Arctic University of Norway, N-9037 Tromso, Norway; ^3^ Department of Oncology, University Hospital of North Norway, N-9038 Tromso, Norway; ^4^ Department of Clinical Pathology, University Hospital of North Norway, N-9038 Tromso, Norway; ^5^ Department of Urology, University Hospital of North Norway, N-9038 Tromso, Norway

**Keywords:** prostate cancer, PDL-1, PD-1, immunohistochemistry, prognostic marker

## Abstract

Programmed cell death protein 1 (PD-1) and its ligand Programmed death ligand 1 (PD-L1) have gained massive attention in cancer research due to recent availability and their targeted antitumor effects. Their role in prostate cancer is still undetermined. We constructed tissue microarrays from prostatectomy specimens from 535 prostate cancer patients. Following validation of antibodies, immunohistochemistry was used to evaluate the expression of PD-1 in lymphocytes and PD-L1 in epithelial and stromal cells of primary tumors. PD-L1 expression was commonly seen in tumor epithelial cells (92% of cases). Univariate survival analysis revealed a positive association between a high density of PD-1+ lymphocytes and worse clinical failure-free survival, limited to a trend (*p* = 0.084). In subgroups known to indicate unfavorable prostate cancer prognosis (Gleason grade 9, age < 65, preoperative PSA > 10, pT3) patients with high density of PD-1+ lymphocytes had a significantly higher risk of clinical failure (*p* = < 0.001, *p* = 0.025, *p* = 0.039 and *p* = 0.011, respectively). In the multivariate analysis, high density of PD-1+ lymphocytes was a significant negative independent prognostic factor for clinical failure-free survival (HR = 2.48, CI 95% 1.12–5.48, *p* = 0.025).

## INTRODUCTION

Prostate cancer is a major contributor to cancer burden and death among men worldwide [1, 2], and issues multiple challenges regarding diagnostics and disease management. There is a lack of molecular markers suitable for determining the prognosis and thus intensity of treatment, resulting in overtreatment with unnecessary side effects on the one hand and undertreatment and disease progression on the other [3]. Once a patient reaches a state of metastatic castration-resistant disease, no curative treatment options are available. Hence, there is an urgent need for new prognostic markers, as well as better treatment options, for both confined and widespread disease in prostate cancer.

It has become evident that for a cancerous tumor to develop and metastasize it has to escape anti-tumor immune response, especially CD8+ cytotoxic T cell mediated elimination [4]. Multiple mechanisms have been identified, including the exploitation of natural immunosuppressive pathways such as the programmed cell death protein 1 (PD-1) pathway [5, 6]. In healthy individuals, this pathway is important for maintaining self-tolerance, as well as curbing T cells during an immune response, preventing collateral damage to healthy tissues [7]. The pathway consists of the receptor programmed cell death protein 1 (known as PD-1 or CD279) and its ligands programmed death ligand 1 (known as PD-L1, CD274 or B7-H1) and programmed death ligand 2 (known as PD-L2, B7-DC or CD273), where the former is believed to be of greatest significance. PD-1 can primarily be found on T cells, but also B cells, Natural Killer T (NKT) cells, activated monocytes, and dendritic cells (DCs) [7]. PD-L1 is typically found on antigen-presenting cells such as macrophages, but can be found on a wide range of cells, including human cancer cells [5, 6]. It is proposed that malignant cells express PD-L1 through genetic mutations or epigenetic changes, and as a response to an inflammatory environment [5]. This enables them to directly inactivate tumor infiltrating lymphocytes (TILs), and hence escaping immune destruction. In addition to activating PD-1, PD-L1 also has immunomodulatory effects within the cell on which it is expressed [5].

Knowledge about the PD-1 pathway's immunosuppressive effects lead to the notion that its inhibition could restore T cell mediated immunity towards tumor cells [8]. Currently, drugs that target PD-1 have been approved by the US Food and Drug Administration (FDA) and European Medicines Agency (EMA) for malignant melanoma and non-small cell lung cancer (NSCLC), and there are currently ongoing trials for drugs targeting PD-L1 [9, 10]. Disappointingly, three recent trials, including a total of 27 patients with metastatic castration-resistant prostate cancer (mCRPC) receiving the PD-1 inhibitor drug nivolumab, demonstrated no clinical benefit [11–13]. In light of the use and development of new PD-1 pathway inhibitors, it is vital to gather information that can shed light on the expression of these immune checkpoint molecules in prostate cancer, and whether their expression is associated with prostate cancer survival. This might aid patient treatment decision-making as well as contributing to future research in PD-1 pathway directed therapies in prostate cancer patients.

Herein, we aimed to examine the potential prognostic significance of PD-1 and/or PD-L1 expression in prostate cancer. Consequently, we investigated 535 primary prostate cancer tumors for expression of PD-L1 in stromal and epithelial cells, as well as the expression of PD-1 and co-expression of PD-1 and CD8 in lymphocytes, and their associations with biochemical and clinical failure-free survival.

## RESULTS

### Patient characteristics and clinicopathological data

Detailed clinical and histopathological characteristics are presented in Table 1. Median age at surgery was 62 (range 47–75). The prostatectomies were retropubic in 435 cases and perineal in 100 cases. At the last follow-up in December 2015, 200 patients had experienced BF, 56 patients had CF, and 18 patients were dead of prostate cancer. Elaborate information on the cohort has been previously published [14].

**Table 1:** Patient characteristics, clinicopathological variables, and molecular markers as predictors of biochemical- and clinical failure in prostate cancer patients (*n* = 535), (univariate analysis; log-rank test) significant *P* values in bold (threshold ≤ 0.05)

Variable	Patients	BF		CF	
	(*n*)	(200 events)		(56 events)	
		5-year EFS (%)	*p*	10-year EFS (%)	*p*
**Age**			0.237		**0.038**
≤ 65 years	357	77		94	
> 65 years	178	70		91	
**pT-stage**			**< 0.001**		**< 0.001**
pT2	374	83		97	
pT3a	114	61		87	
pT3b	47	43		74	
**Preop PSA**			**< 0.001**		**0.029**
PSA<10	308	81		95	
PSA>10	221	68		89	
Missing	6	-		-	
**Gleason grade**			**< 0.001**		**< 0.001**
3+3 / Grade group 1	183	83		98	
3+4 / Grade group 2	219	77		94	
4+3 / Grade group 3	81	70		90	
4+4 / Grade group 4	17	58		86	
>8 / Grade group 5	35	37		65	
**Tumor Size**			**< 0.001**		**0.002**
0-20 mm	250	83		96	
>20 mm	285	68		90	
**PNI**			**< 0.001**		**< 0.001**
No	401	80		96	
Yes	134	60		83	
**PSM**			**0.049**		0.198
No	249	69		90	
Yes	286	81		96	
**Non-apical PSM**			**< 0.001**		**< 0.001**
No	381	82		96	
Yes	154	57		85	
**Apical PSM**			0.063		0.427
No	325	74		92	
Yes	210	77		93	
LVI			**< 0.001**		**< 0.001**
No	492	77		95	
Yes	43	47		69	
**Surgical proc**			0.466		0.308
Retropubic	435	77		92	
Perineal	100	68		95	
**PD-1+ lymphocytes in TS**			0.489		0.084
Low	353	74		94	
High	43	69		87	
Missing	139				
**PD-L1+ TS cells**			0.899		0.680
Low	245	28		92	
High	157	74		91	
Missing	133				
**PD-L1+ TE cells**			0.078		0.603
Low	166	77		92	
High	236	71		92	
Missing	133				

Abbreviations: BF = biochemical failure; CF = clinical failure; EFS = event free survival in months; LVI = lymphovascular infiltration; p = *p* value for difference in event free survival with log rank analysis; PD-1 = programmed cell death protein 1; PD-L1 = programmed death-ligand 1; PNI = Perineural infiltration; Preop = preoperative; PSA = Prostate specific antigen; PSM = Positive surgical margin; pT-stage = pathological tumor stage; Proc = procedure; TE = tumor epithelial cells; TS = tumor stromal cells

### Programmed cell death protein 1 and programmed death ligand 1 expression in prostate tumor tissue

**Figure 1:**
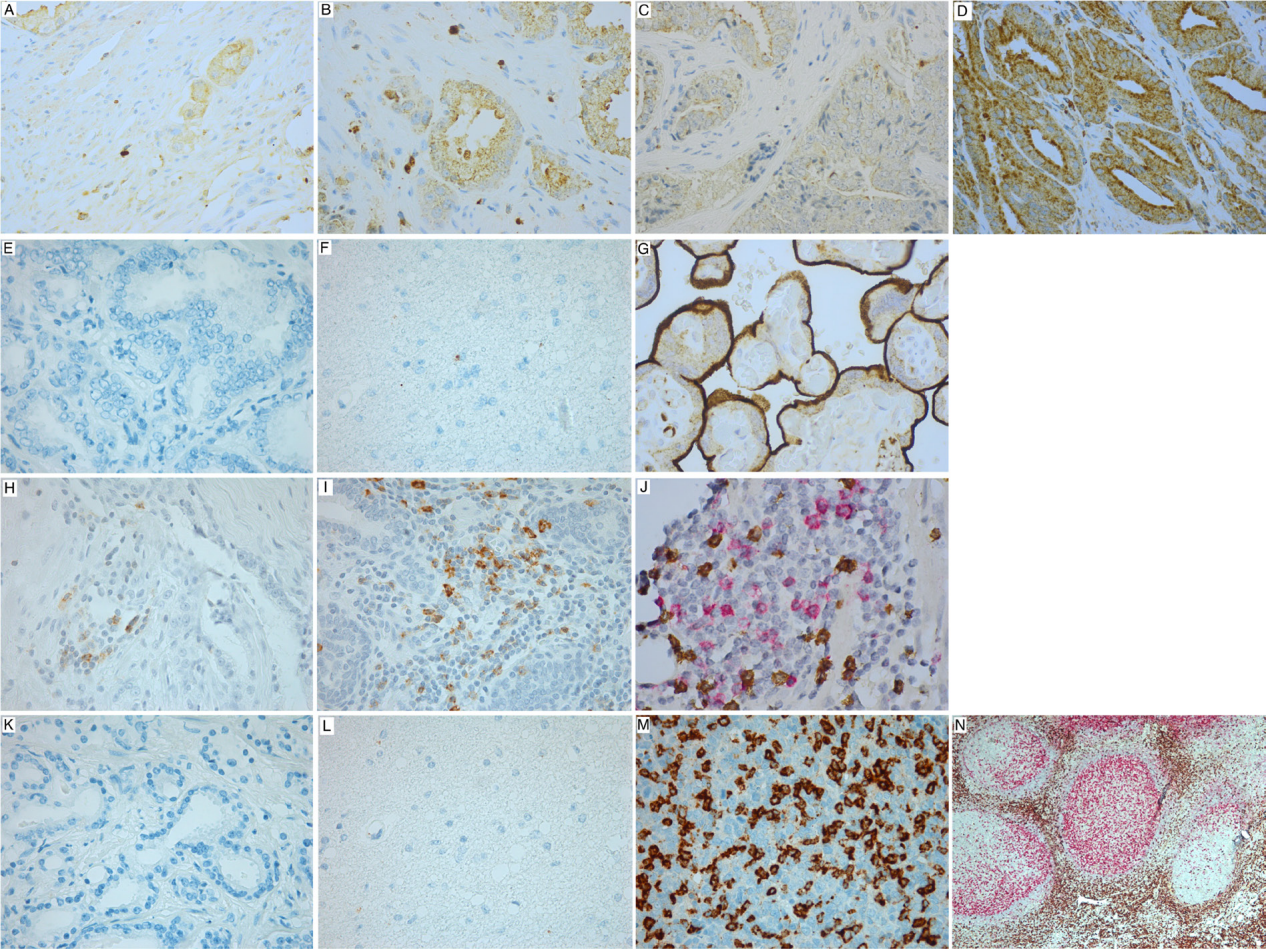
Immunohistochemical analysis (**A**) Low density PD-L1+ stromal cells, (**B**) High density PD-L1+ stromal cells, (**C**) Low intensity PD-L1+ tumor epithelial cells, (**D**) High intensity PD-L1+ tumor epithelial cells, (**E**) Negative isotype control antibody for PD-L1 (prostate TMA), (**F**) Negative control for PD-L1 (brain), (**G**) Positive control for PD-L1 (placenta), (**H**) Low density of intratumoral PD-1+ lymphocytes, (**I**) High density of intratumoral PD-1+ lymphocytes, (**J**) PD-1 and CD8 double stain with pink showing PD-1 positivity, and brown showing CD8 positivity, (**K**) Negative isotype control antibody for PD-1, (**L**) Negative control for PD-1 (brain), (**M**) Positive control for PD-1 (tonsil), (**N**) Positive control for PD-1 and CD8 double stain (tonsil). Magnification ×400 for all, except (**N**) which shows ×50 magnification.

Of the total cohort of 535 patients, immunohistochemistry (IHC) tumor scoring was possible for 402 cases for PD-L1, and 396 for PD-1. PD-L1 expression (Figure 1) was both cytoplasmatic and membranous. Intraluminal secretions and some intracellular granules seemed to stain intensively and were disregarded as artifacts. PD-L1 staining in tumor epithelial (TE) cells was positive in 371/402 (92%) cases, and 236/402 (59%) cases had a high PD-L1 intensity score. In addition, 267/402 (66%) of patients had PD-L1+ stromal cells. In general, PD-1+ cells were sparse (Figure 1) and fit the morphology of lymphocytes. In total, 156/396 (39%) cases had such intratumoral PD-1+ lymphocytes, and 43/396 (11%) cases had a high density. In addition, we observed few intraepithelial PD-1+ cells. Some of these resembled tumor cells, as recently described for malignant melanoma [15]. Unfortunately, we were not certain these were tumor cells using only morphological assessment, and this, in addition to low numbers, made them impossible to quantify by scoring. CD8 and PD-1 double staining showed co-expression of CD8 and PD-1, but also lymphocytes with single expression of one marker (Figure 1). However, the brown CD8 staining overpowered the red stain of PD-1, making quantification by scoring difficult.

### Correlations between programmed cell death protein 1, programmed death ligand 1, lymphocyte markers and clinicopathological variables

The expression of PD-L1+ tumor stromal (TS) cells correlated significantly with PD-L1+ TE cells (r = 0.36, *p* = < 0.001), and had a weak correlation with intratumoral PD-1+ lymphocytes (*r* = 0.21, *p* = < 0.001). The expression of PD-L1 in TE cells and TS cells, in addition to intratumoral PD-1+ lymphocytes did not correlate to previously published [16] tumor tissue expression of lymphocyte markers CD3, CD4, CD8 and CD20. The expression of PD-1+ lymphocytes and PD-L1 in TS and TE was not correlated to clinicopathological variables (age, pT stage, preoperative PSA, Gleason grade, tumor size, perineural infiltration, lymphovascular infiltration and non-apical positive surgical margin).

### Univariate survival analysis

**Figure 2:**
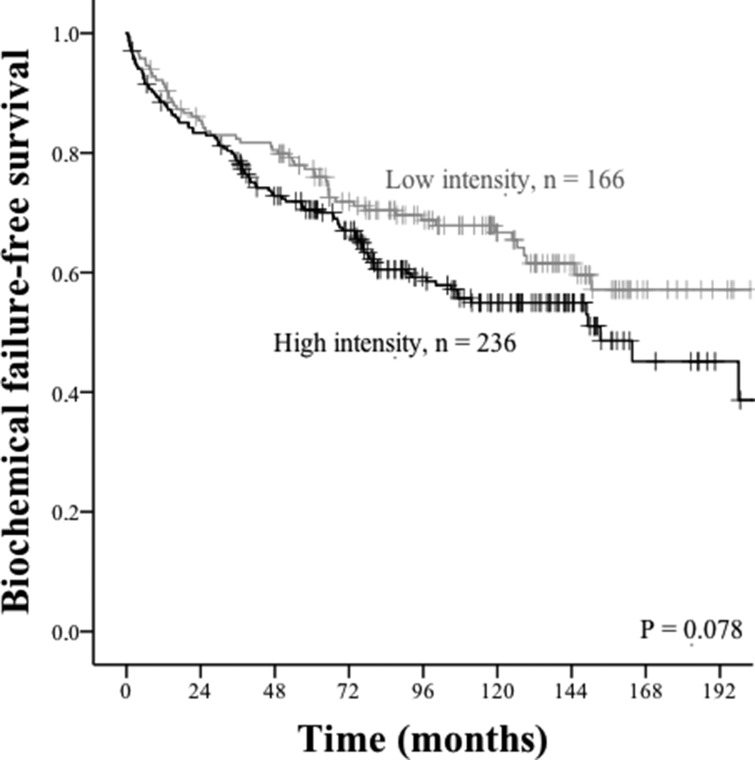
Biochemical failure-free survival curves for PD-L1 intensity in tumor epithelial cells Grey lines indicate low intensity, whereas black lines indicate high intensity.

**Figure 3:**
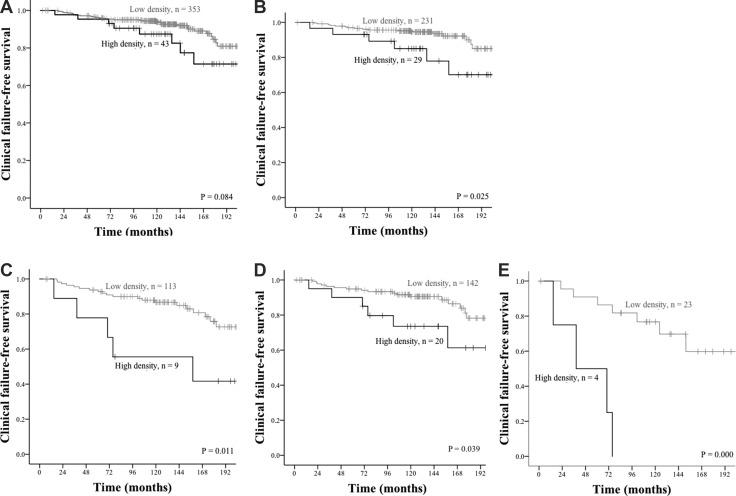
Clinical failure-free survival curves for PD-1+ lymphocytes in tumor stromal areas Grey lines indicate low density, whereas black lines indicate high density. (**A**) All patients, (**B**) Patients with age < 65, (**C**) Patients with pTstage = 3, (**D**) Patients with preoperative PSA > 10, (**E**) Patients with Gleason grade = 9.

The results of the univariate survival analyses are presented in Table 1 and Figures 2 and 3. Neither PD-L1+ TE cells nor PD-L1+ TS cells reached statistical significance for predicting biochemical failure (BF) or clinical failure (CF), but there was a trend towards a negative association between PD-L1 expression in TE cells and outcome, most prominently for biochemical failure-free survival (BFFS) (HR: 1.34 (CI95% 0.97–1.85) *p* = 0.078, Table 1, Figure 2). With regard to PD-1+ lymphocytes, there was a trend for worse clinical failure-free survival (CFFS) in the entire patient material (HR: 1.96 (CI95% 0.90–4.25), *p* = 0.084, Table 1, Figure 3), but subgroups known to indicate unfavorable prostate cancer prognosis had a significantly higher risk for CF if they had a high density of intratumoral PD-1+ lymphocytes: age < 65 (*p* = 0.025), pT3 stage (*p* = 0.011), preoperative PSA > 10 (*p* = 0.039), and Gleason grade 9 (*p* = < 0.001) (Figure 3).

### Multivariate survival analysis

Clinicopathological variables and PD-1 and PD-L1 variables with *p* < 0.10 from the univariate analyses (Table 1) were entered into three different multivariate models and results are presented in Table 2. High expression of intratumoral PD-1+ lymphocytes was a significant negative independent prognostic factor for CFFS (HR = 2.48, CI95% 1.12–5.48), *p* = 0.025) together with Gleason grade and perineural infiltration.

**Table 2:** Independent predictors for biochemical- and clinical failure in prostate cancer patients (*n* = 535), (cox regression analysis, backward conditional model)

Variable	Model 1 (clinicopathological)				Model 2 (PD-L1+ TE)		Model 3 (PD-1+ lymphocytes in TS)	
	BF		CF		BF		CF	
	HR (95% CI)	*p*	HR (95% CI)	*p*	HR (95% CI)	p	HR (95% CI)	*p*
***Age***	NE		NS		NE		NS	
≤ 65 years								
> 65 years								
***pT-stage***		***0.001***	NS			***0.003***	NS	
pT2	1.00				1.00			
pT3a	1.48 (1.02–2.14)	***0.040***			1.50 (1.00–2.27)	***0.050***		
pT3b	2.34 (1.47–3.74)	***< 0.001***			2.41 (1.45–4.00)	***0.001***		
***Preop PSA***		***0.033***	NS		NS		NS	
PSA < 10	1.00							
PSA > 10	1.37 (1.03–1.84)							
***Gleason grade***		***0.040***		***< 0.001***		***0.011***		***< 0.001***
3 + 3/Grade group 1	1.00		1.00		1.00		1.00	
3 + 4/Grade group 2	1.24 (0.86–1.78)	0.249	3.74 (1.40–9.98)	***0.009***	1.02 (0.67–1.56)	***0.920***	4,70 (1,31–16,81)	***0.017***
4 + 3/Grade group 3	1.73 (1.12–2.68)	***0.013***	5.08 (1.73–14.88)	***0.003***	1.98 (1.21–3.25)	***0.007***	6,26 (1,66–23,63)	***0.007***
4 + 4/Grade group 4	2.13 (1.06–4.31)	***0.035***	5.95 (1.41–25.14)	***0.015***	2.05 (0.96–4.37)	0.063	10,10 (2,04–50,17)	***0.005***
> 8/Grade group 5	1.92 (1.09–3.39)	***0.025***	13.09 (4.46–38.40)	***0.000***	1.83 (1.00–3.36)	***0.050***	20,34 (5,71–72,48)	***< 0.001***
***Tumor size***	NS		NS		NS		NS	
0–20 mm								
> 20 mm								
***PNI***		***0.045***	NS			***0.017***		***0,012***
No	1.00				1.00		1.00	
Yes	1.40 (1.01–1.94)				1.56 (1.08–2.25)		2,32 (1,21–4,47)	
***Non-apical PSM***		***0.001***	NS			***0.026***	NS	
No	1.00				1.00			
Yes	1.73 (1.25–2.38)				1.50 (1.05–2.14)			
***Apical PSM***		***0.026***	NE		NS		NE	
No	1.00							
Yes	0.71 (0.52–0.96)							
***LVI***	NS		NS		NS		NS	
No								
Yes								
***PD-L1 + TE cells***	NE		NE		NS		NE	
Low								
High								
***PD-1 + lymphocytes in TS***	NE		NE		NE			***0.025***
Low							1.00	
High							2.48 (1.12–5.48)	

Abbreviations: BF = biochemical failure; CF = clinical failure; CI = confidence interval; HR = hazard ratio; LVI = lymphovascular infiltration; NE = not entered in analysis; NS = not significant; p = *p* value for difference in survival with Cox regression analysis; PD-1 = programmed cell death protein 1; PD-L1 = programmed death-ligand 1; PNI = Perineural infiltration; Preop = preoperative; PSA = Prostate specific antigen; PSM = Positive surgical margin; pT-stage = pathological tumor stage; TE = tumor epithelial cells; TS = tumor stromal cells

## DISCUSSION

In our large, multicenter cohort of 535 prostate cancer cases, we observed a high density of PD-1+ lymphocytes in prostate cancer tumor tissue to independently predict shorter CFFS. The prognostic impact of PD-1 was stronger than for any of the renowned clinicopathological features, except for Gleason grade. In addition, a high density of PD-1+ lymphocytes was significantly associated with shorter CFFS in most subgroups related to worse prostate cancer prognosis, such as low age, high pT-stage, high preoperative PSA, and high Gleason grade. Furthermore, 92% of the cases had some level of PD-L1 expression in TE cells, but no significant association between the marker and outcome was observed.

To our knowledge, this is the first study to examine the prognostic impact of both PD-1 and its ligand PD-L1 in the same prostate cancer cohort, and the first to explore prognostic effects of PD-1+ lymphocytes in prostate cancer altogether. In addition to novelty, two major strengths in our study are the large, unselected patient population and the long follow-up time enabling us to calculate prognoses with regard to relevant clinical endpoints. Since no antibody for quantifying PD-1, and especially PD-L1, in formalin-fixed paraffin embedded (FFPE) tissue is uniformly accepted as standard, the antibodies used herein underwent stringent confirmatory validation in our laboratory, in addition to the manufacturers in-house validation.

This study was conducted as a further elaboration of our previous observation that CD8+ lymphocytes are independent negative prognostic markers in prostate cancer [16]. Based on this discovery, we proposed that the detected CD8+ lymphocytes were indeed tumor-specific CD8+ T cells summoned to particularly aggressive tumors, but lacking functionality due to immunosuppression, for example due to activation of the PD-1 pathway. The CD8-marker represents a broad population of T cells with various roles, which might weaken its prognostic impact. A recent study in melanoma patients concluded that PD-1 expression on CD8+ T cells identifies the subpopulation of tumor-specific effector cells [17] and hence, PD-1 may be a more specific prognostic marker than CD8. Surprisingly, our previously published lymphocyte marker expressions (CD3, CD4, CD8 and CD20) [16] did not correlate with PD-1 expression. To further explore the relationship between CD8+ and PD-1+ cells, we double-stained for both markers. By microscopic examination we detected co-expression of CD8 and PD-1 on lymphocytes as suspected, but also cells with single PD-1 or CD8 marker expression. Another explanation for lack of statistical correlations may be the different scoring methods of lymphocyte markers and PD-1 [16]. Also limiting the ability for comparison, the current study was performed on TMA cores cut from a much deeper tissue level than the lymphocyte study [16] hampering the ability to correlate these markers in the same tissue areas.

So far, translational studies regarding the prognostic impact of PD-1 and PD-L1 in human prostate cancer are sparse. For PD-1, only descriptive analyses have been published, all reporting PD-1+ lymphocytes to be present in prostate cancer carcinoma regions and/or in adjacent TLS [18–20]. With regard to PD-L1 positive tumor epithelial cells, descriptive analyses have been conflicting: Some research groups have reported lack of tumor epithelial positivity [12, 13, 20], while others have observed sparse expression [21] or cases with high expressions [18]. In a recent study, Gevensleben et al. found a high PD-L1 expression in TE cells to be an independent negative prognostic factor of BFFS in a cohort of 902 men with prostate cancer [22]. We could not fully reproduce this result, but in both univariate and multivariate analyses there was a consistent tendency of high expression of PD-L1 in TE cells in patients with a worse BFFS. In a larger study population, this association may have reached statistical significance.

There may be multiple possible biological explanations as to why we and others [18, 22] observe such high expression levels of PD-L1 in prostate tumor epithelial cells. The most well-known mechanism of PD-L1 induction on tumor epithelial cells is cytokines such as IFNγ produced by adaptive immune cells in the tumor microenvironment (‘adaptive immune resistance’) [21, 23, 24]. However, we find that PD-L1 expression do not correlate to adaptive immune cell markers, which may suggest there is another mechanism at play. Several studies in different cancers have demonstrated that intrinsic oncogenic pathways may induce PD-L1 expression (‘innate immune resistance’). Some examples include EGFR mutations [25, 26] and loss of phosphatase and tensin homolog (PTEN) [27, 28]. To our knowledge, no such relationships have been found between intrinsic pathways and PD-L1 expression in human prostate cancer [21].

Our finding that an augmentation of the PD-1 pathway leads to a worse prostate cancer prognosis may indicate that tumor immune escape, and thus tumor immune elimination, are important mechanisms in prostate cancer. CD8+ cytotoxic T cells are proposed to be one of the most important protagonists in tumor immune elimination, and the mechanisms by which tumor cells avoid attack by tumor-specific CD8+ T cells are crucial parts of the immune escape process [4]. Different escape routes have been proposed. FOXP3+CD25+CD4+ Tregs are known suppressors of CD8+ cytotoxic T cells, and are observed up-regulated in multiple cancer types, including prostate cancer [29–31]. In addition, the process of antigen presentation is often impaired in tumors, leading to inadequate activation and boosting of T cells [32]. Moreover, tumor-specific CD8+ T cells have been found to express exhaustion markers such as PD-1 and T-cell immunoglobulin and mucin-domain containing-3 (Tim-3) indicating that their presence not necessarily implies an effective ongoing immune elimination process [33–35]. Contributing to this, different tumor cells have been found to express molecules such as indoleamine-2,3-dioxygenase (IDO) [36] and PD-L1, known to impair function of CD8+ cytotoxic T cells [5].

There have been recent breakthroughs in PD-1 pathway inhibition in other cancer diseases [9]. Our study has found the pathway molecules to be present in prostate cancer, and their presence to be associated to poor prognosis and as such, proposing them as attractive targets for inhibition. However, results from prostate cancer studies have so far proven mostly disappointing. At the time we conducted this study there had been published results from three different clinical trials with a total of 27 prostate cancer patients treated with the PD-1 inhibitor nivolumab [11–13]. Unfortunately, no clinical benefits were observed for these cases, which may have several possible explanations. For an immune checkpoint inhibitor to be effective in cancer treatment, the cancer in question must be able to evoke an immune response. Thus, one possible reason why PD-1 blockage does not appear to work in prostate cancer, may be that it is not an immunogenic cancer type. However, there are several aspects contradicting this proposition. Firstly, prostate cancer can express multiple tumor-associated antigens necessary in triggering anti-tumor immune response [such as prostate-specific antigen (PSA), prostatic acid phosphatase (PAP), prostate-specific membrane antigen (PSMA), and prostate stem cell antigen (PSCA)] [37, 38]. Secondly, the FDA approved autologous dendritic cell-based vaccine Sipuleucel-T extends survival in patients with mCRPC. Though its exact mechanisms are not known, the most likely explanation is that it generates a tumor-specific T cell mediated immune response [31]. Furthermore, the mechanisms by which anti-androgen treatment increases survival in prostate cancer are believed to be partly explained by its ability to boost a tumor specific immune response [38, 40–41].

A likely reason why nivolumab-trials have failed to show effect in prostate cancer patients may be differences in patient and tumor characteristics. Patients in both our and Gevensleben et al. [22] cohorts were hormone naïve while the mentioned nivolumab trials [11–13] only included patients with mCRPC. Hence, immunosuppression through the PD-1 pathway may be a less efficient mechanism in late stage, widespread cancer disease, and/or there may be a more direct relationship between androgens and the PD-1 pathway. In addition, all prostate cancer patients included in the nivolumab-studies was reported to be negative for tumor expression of PD-L1 (< 5% PD-L1 positive cells) [12, 13]. As no prostate cancer patients with a high degree of PD-L1 positive tumor cells have received nivolumab, the trials give no genuine data on the efficacy of PD-1 pathway inhibitor treatment. However, we have, corroborating others [22], demonstrated that such expression is common in primary prostate cancer tumors from patients with localized disease. Though there is an ongoing debate regarding whether PD-L1 tumor expression can predict treatment effect, there are multiple indications that PD-L1 positivity enrich for response to PD-1 pathway inhibitors [43]. Hence, PD-1 pathway inhibitors should not be completely disregarded as ineffective in prostate cancer treatment, and in a recent trial with pembrolizumab 3/10 patients had an almost complete PSA regression [44].

To conclude, we find PD-1+ lymphocytes in prostate cancer tumors to be an independent negative prognostic marker in post-prostatectomy hormone naïve patients. In addition, our observations imply that PD-1 pathway inhibitors may yield therapeutic benefit in selected groups of prostate cancer patients.

## MATERIALS AND METHODS

### Patient characteristics and clinicopathological data

Six hundred and seventy-one patients who underwent radical prostatectomy as initial treatment for prostate adenocarcinoma from 1995 to 2005 were retrospectively identified from the Departments of Pathology at the University Hospital of Northern Norway (*n* = 267), Nordland Hospital (*n* = 63), St. Olavs Hospital (*n* = 330), and Levanger Hospital (*n* = 11). One hundred and thirty-six patients did not meet the inclusion criteria due to: (i) radiotherapy to the pelvic region prior to surgery (*n* = 1), (ii) other malignancies within 5 years prior to the prostate cancer diagnosis (*n* = 4), (iii) inadequate paraffin-embedded tissue blocks (*n* = 130), and (iv) lack of follow-up data (*n* = 1). Thus, a total of 535 patients were included in this study. Complete demographic and clinical data were obtained from medical records. Two experienced pathologists (ER and LTB) reviewed all cases and registered histopathological data. Tumors were histologically classified according to WHO guidelines [45], graded in accordance with both the modified Gleason grading system [46, 47] and the new contemporary Gleason grading system [48], and staged in agreement with International Union Against Cancer (UICC) guidelines [49]. All demographic-, clinical- and histopathological data (Table 1) were registered in a SPSS data file and patients were de-identified. This report includes follow-up data as of December 2015. Median follow-up of survivors was 150 (range 17–245) months. For extensive information regarding our cohort, see our previous report [14]. The ethics of this study has been approved by The Regional Committees for Medical and Health Research Ethics (Protocol ID: 2009/1393, extended approval 2015), The Norwegian Data Protection Authority, and The Data Protection Official for Research (The Norwegian Social Science Data Service). Informed consent was not obtained, but the data was analyzed de-identified and this report contains no identifiable details.

### Tissue microarray construction

For each case, a pathologist (ER) histologically identified and marked separate areas of the most representative TE tissue, adjacent TS tissue, normal epithelial (NE) tissue, and normal stromal (NS) tissue. In brief, a tissue-arraying instrument (Beecher Instruments, Silver Springs, MD) with a 0.6 mm diameter needle was used to harvest a total of 6 cores from each case from the corresponding paraffin-embedded tissue blocks. The samples were inserted into a recipient paraffin block, and from each block 4 μm sections were cut with a Micron microtome (HM355S), affixed to glass slides, and sealed with paraffin.

### Validation of antibody specificity

The primary antibodies used in this study were as follows: (i) PD-L1 rabbit monoclonal antibody (Cat#13684, clone: E1L3N, Cell Signaling Technology, Danvers, MA, USA), (ii) PD-1 mouse monoclonal antibody (Cat#ab52587, clone: NAT105, Abcam, Cambridge, UK) and (iii) CD8 rabbit monoclonal antibody (clone SP57; Ventana; Cat#790-4460). All applied antibodies had been subjected to in-house validation by their manufacturer. In addition, we performed confirmatory validation for PD-L1 and PD-1 to further accredit antibody specificity. Overexpressed human HEK293T cell lysates were utilized from OriGene for PD-L1 (#LY415473), PD-1 (#LY401555) and HEK293 as empty vector (#LY500001/negative control). Cells were incubated with 2xSDS sample buffer (OriGene) for 10 minutes at 100°C. Equal amounts of protein lysates were resolved on to a 4 to 12% Bis-Tris gel (Cat#NP0322; Life Technologies), and transferred onto an Odyssey nitrocellulose membrane (Cat#926-31092, LI-COR). The membrane was subsequently blocked for 1 hour at room temperature using Odyssey blocking buffer (Cat#927-40000, LI-COR). For PD-L1 1/1000, and for PD-1 1/50 dilution of primary antibody was applied and the membrane incubated overnight at 4°C. PD-L1 (Cat#926-32213, LI-COR), and PD-1 (Cat#926-32212, LI-COR) RDye 800CW secondary antibodies with 1:1000 dilution was then applied, and the membrane incubated 1 hour at room temperature. Between antibody incubations, the membrane was washed 3 times for 5 minutes, each time in tris-buffered saline containing 0.05% Tween 20 (Cat#T9039, Sigma-Aldrich). Molecular weight markers used were the MagicMark XP Western Protein Standard (Cat#LC5603, Invitrogen) and SeeBlue Plus2 Pre-stained Standard (Cat#LC5925, Invitrogen). The most prominent bands (Supplementary Figure 1) represent the observed molecular weight of the detected protein, which correspond intimately with the predicted weight. Rabbit anti-actin, diluted 1:1000 (Cat#A2066, Sigma-Aldrich) was used as internal control and all lanes showed 42 KDa molecular weight protein load as predicted (Supplementary Figure 1).

### Immunohistochemistry

Prior to IHC analysis, all slides were heated at 60°C for tissue fixation. PD-L1 IHC was performed on a Discovery-Ultra immunostainer (Ventana Medical Systems, Tucson, AZ). Slides were deparaffinized on-board in three 8-minute cycles. Antigen retrieval was done by using the EDTA-based solution (pH 8.0–8.5) CC1 reagent (Cat#950-124) at 95°C and incubating for 64 minutes. Endogenous peroxidase was blocked by incubating with Discovery inhibitor (Cat#760-4840) for 8 minutes. Primary antibody PD-L1 with 1/25 dilution was added and slides were incubated for 32 minutes at 37°C. Secondary antibody used was UltraMap anti-rabbit HRP (Cat#760-4315) incubating for 20 minutes, followed by 8 minutes HRP amplification. Finally, ChromoMap DAB (Cat#760-159) was used to visualize the antigens.

PD1/CD8 dual and PD1 IHC were performed using the Ventana Benchmark XT automated immunostainer (Ventana Medical Systems, Tucson, AZ). Antigen retrieval was done for 30 minutes at approximately 100°C with CC1 reagent (Cat#950-124). Primary CD8 prediluted antibody was incubated for 12 minutes and visualized using the polymer-based Ventana ultraView DAB detection kit (Cat#760-500). The protocol followed by an ultraWash step to wash off excess antibody. Antibody denaturation for 4 minutes at 90°C was performed to ensure that the first primary antibody was completely inactivated before applying the second primary antibody. The PD-1 primary antibody in a 1/50 dilution was incubated for 32 minutes. The primary antibody was visualized using the Ventana ultraView Universal Alkaline Phosphatase Red Detection Kit (Cat#760-501) for double stain. The single staining of PD-1 was performed with same antigen retrieval procedure and was visualized with ultraView DAB detection kit.

To visualize the nuclei, all slides were counterstained with Ventana Hematoxylin II reagent (Cat# 790-2208) for 32 minutes, followed by a Bluing reagent (Cat# 760-2037) for 8 minutes, and then dehydrated, cleared and mounted on glass slides. All double stained sections were compared with the corresponding single stained slide. Two different controls were applied. First, control staining with an isotype-matched control antibody without the primary antibody, under the same staining protocol as for the primary antibody. Rabbit and mouse isotype-matched negative control antibodies were obtained from Abcam (PD-L1, Cat#ab27478; PD-1, Cat#ab18443). Second, multiple organ TMA as positive and negative tissue controls were used to verify the specificity of the staining in every staining procedure. The positive tissue controls comprised placenta for PD-L1 and tonsil for PD-1 (Figure 1). Negative tissue controls were samples of normal brain and ventricle for both PD1 and PD-L1.

To confirm staining homogeneity of PD-L1 throughout the tumor epithelium, we selected six patients from TMA slides with different tumoral expression (low, moderate, high). Whole tissue sections of these patients were further stained with PD-L1 (Cat#13684, clone: E1L3N), and analyzed by an experienced pathologist who approved staining homogeneity (Supplementary Figure 2).

### Scoring of immunohistochemistry

All tissue samples were scored semi-quantitatively by two investigators independent of each other, and blinded to clinicopathological data and patient outcome. PD-L1 was scored by two experienced pathologists (AV, CN) and PD-1 was scored by one experienced pathologist (ER) and one trained MD (NN). For each tissue core the most experienced pathologist histologically assured the tissue type, and if possible 2 cores of TE, 2 of TS, 1 of NE and 1 of NS was scored for each case. Because PD-L1 was uniformly homogenously expressed in epithelial cells, an intensity scoring scale was chosen, and were as follows: no staining = 0, weak staining = 1, moderate staining = 2, and strong staining = 3. PD-L1+ stromal cells and PD-1+ lymphocytes were scored as number of positive stained cells per 0.6 mm diameter core as follows: 0 = 0–3, 1 = 4–10, 2 = 11–15, and 3 = > 15. In case of major disagreement (scoring difference > 1), the core was re-examined and consensus was reached. For each case, the mean score was calculated for each tissue compartment, and further dichotomized into low and high expression. Cut-off values for dichotomization were chosen according to a minimal *P-value* approach (optimal cut-off) while also securing statistically sufficient numbers in each group, and high scores were defined as follows: (i) ≥ 0.54 (mean) for PD-L1+ TS cells (ii) ≥ 1.0 for PD-L1 TE cells, and (iii) ≥1.25 for intratumoral PD-1+ lymphocytes. Scoring agreement between investigators was excellent for both markers. The intra-class correlation coefficient (reliability coefficient, r) was 0.93 (CI95% 0.92–0.93, *p* < 0.001) for PD-L1 and 0.96 for PD-1 (CI95% 0.57–0.96, *p* < 0.001). Slides with CD8 and PD-1-double staining were examined, but not quantified by scoring.

### Statistical analysis

All statistical analyses were performed using the statistical package IBM SPSS, version 23 (SPSS Inc., Chicago, IL). The IHC scoring values from each observer were compared for inter-observer reliability by use of a two-way random effect model with absolute agreement definition. Spearman's rank-correlation was used to examine the associations between PD-L1 and PD-1 expressions, previously published lymphocyte markers [15] and clinicopathological markers. Presented r-values are the Spearman's rank correlation coefficient. Univariate Cox regression analysis was used to generate HR for each individual variable. Univariate survival analyses were done by using the Kaplan–Meier method, and the difference between survival curves was assessed by the log-rank test. The survival curves were terminated at 192 months, as less than 10% of patients were at risk after this point. All significant variables from the univariate analyses were assessed in multivariate survival models using a backward stepwise Cox regression model with a probability for stepwise entry or removal at *p* = 0.05 and 0.10, respectively. The significance level was *p* < 0.05 for all analyses. All survival analyses were carried out using BF and CF as endpoints. BF was characterized as a PSA ≥ 0.4 ng/ml, and rising in a minimum of two different blood samples postoperatively. BFFS was calculated as time from surgery to last follow-up date or the date PSA was first measured above threshold. CF was defined as verified local, symptomatic cancer recurrence and/or radiological verified metastasis to bone, visceral organs or lymph nodes after prostatectomy. CFFS was calculated from date of surgery to last follow-up date without CF or to date of CF.

## SUPPLEMENTARY MATERIALS FIGURES


