# Supplementary material for: The prognostic role of immune checkpoint markers programmed cell death protein 1 (PD-1) and programmed death ligand 1 (PD-L1) in a large, multicenter prostate cancer cohort
Source: Oncotarget. 2017 Mar 1;8(16):26789–801. doi: 10.18632/oncotarget.15817 (PMC5432297; doi:10.18632/oncotarget.15817)
Supplement: Supplementary file 1 [file oncotarget-08-26789-s001.pdf]

The prognostic role of immune checkpoint markers programmed cell death protein 1 (PD-1) and programmed death ligand 1 (PD-L1) in a large, multicenter prostate cancer cohort

Supplementary Materials

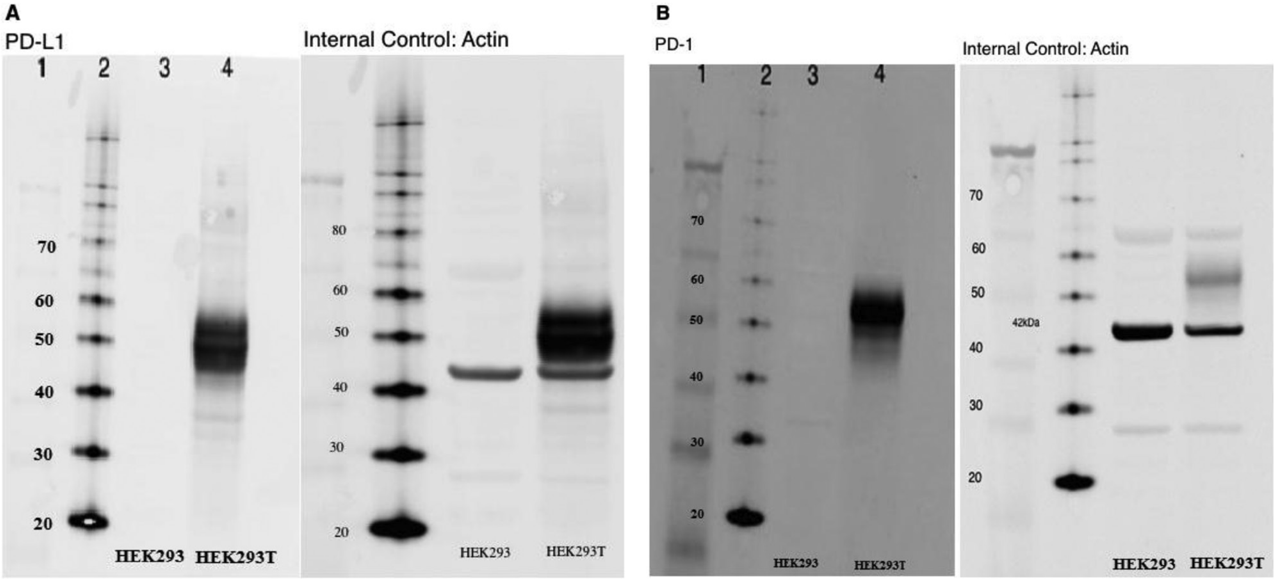

Supplementary Figure 1: Antibody validation. (**A**) PD-L1 (Cat#13684, clone: E1L3N, Cell Signaling Technology, Danvers, MA, USA) and (**B**) PD-1 (Cat#ab52587, clone: NAT105, Abcam, Cambridge, UK). 1 and 2, Molecular weight markers (1, SeeBlue Plus2 Pre-Stained and 2, Magic Marker XP Western Protein); 3, Empty vector (#LY5000001/negative control); 4, Transient overexpressed human HEK293 T cell lysates for PD-L1 (#LY415473) and PD-1 (#LY401555). The most prominent bands represent the observed molecular weight of the detected protein, which corresponded intimately with the predicted weight provided by the manufacturer.

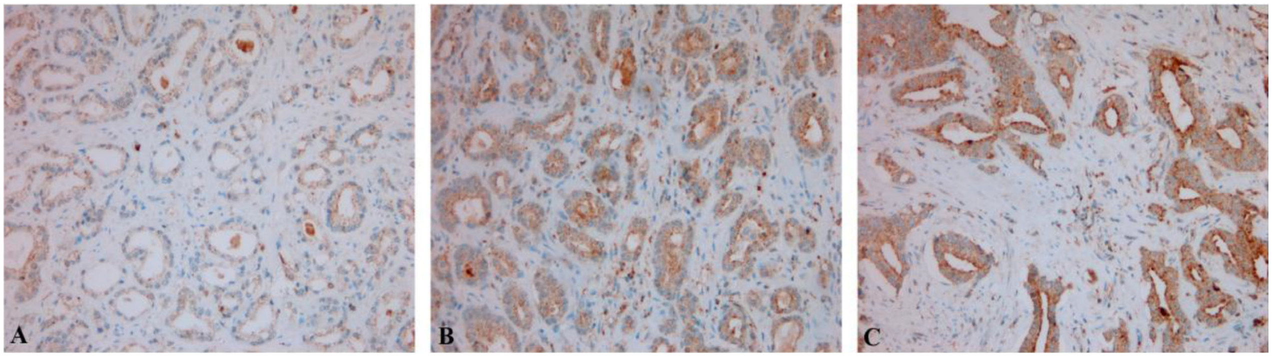

Supplementary Figure 2: Photomicrographs of PD-L1 expression in whole tissue section-slides. Validation of TMA and staining homogeneity of PD-L1 throughout tumor epithelium using whole sections. (**A**) Low, (**B**) moderate and (**C**) high intensity of tumor epithelium PD-L1, matched in scale with corresponding TMA cores. Magnification ×200.
